# Histone deacetylation links jasmonic acid signaling to flower size control in rose

**DOI:** 10.1186/s43897-026-00261-8

**Published:** 2026-09-04

**Authors:** Yufei Chang, Jun Lu, Yali Li, Xin Jin, Chunguo Fan, Hmmam Zarif, Guozhen Yuan, Rui Zhou, Hongchi Liu, Jingjing Sun, Changquan Wang, Jinyi Liu

**Affiliations:** College of Horticulture, Nanjing Agricultural University, Nanjing, 210095 China

**Keywords:** *Rosa chinensis*, Jasmonic acid, *RcMYC2*, Histone deacetylation, Flower size

## Abstract

Flower size is a key ornamental trait in roses (*Rosa* spp.), determined by coordinated regulation of cell proliferation and expansion. Although jasmonic acid (JA) influences plant growth, its role in flower size control remains unclear. Here, we showed that methyl jasmonate (MeJA) treatment reduces flower size in *Rosa chinensis*, accompanied by elevated expression of the JA-responsive transcription factor *RcMYC2*. Silencing *RcMYC2* resulted in larger flowers due to enhanced petal cell expansion and upregulation of cell expansion-related genes. Mechanistically, RcMYC2 directly bound to the promoter of the expansin gene *RcEXPA8* and repressed its transcription via its interaction with the Mediator subunit RcMED25. Consistently, MeJA failed to reduce flower size in *RcMED25*-silenced plants. Upon JA signal activation, RcMYC2 recruited RcMED25 and the histone deacetylase RcHDA8 to the *RcEXPA8* promoter, leading to transcriptional repression via histone deacetylation and restriction of petal cell expansion. These findings demonstrate that histone deacetylation links JA signaling to flower size control, providing new insights into hormone-mediated regulation of organ growth in ornamental plants.

**Supplementary Information:**

The online version contains supplementary material available at https://doi.org/10.1186/s43897-026-00261-8.

## Core

This study reveals a jasmonic acid (JA)-mediated epigenetic mechanism controlling rose flower size. The transcription factor RcMYC2 interacts with Mediator subunit RcMED25 and recruits histone deacetylase RcHDA8 to repress expansin gene RcEXPA8 transcription via histone deacetylation, thereby restricting petal cell expansion and reducing flower diameter.

## Gene & accession numbers

The GenBank (https://lipm-browsers.toulouse.inra.fr/pub/RchiOBHm-V2/) accession numbers for rose genes used in this study are listed as follows: *RcMYC2* (RchiOBHm_Chr7g0209751), *RcMED25* (RchiOBHm_Chr4g0435671), *RcEXPA8* (RchiOBHm_Chr5g0072761), *RcHDA1/19* (RchiOBHm_Chr6g0281731), *RcHDA3* (RchiOBHm_Chr2g0151281), *RcHDA6* (RchiOBHm_Chr7g0192801), *RcHDA8* (RchiOBHm_Chr5g0048241), *RcHDA14* (RchiOBHm_Chr2g012185).

## Introduction

Flower size is an ecologically and economically important trait in many flowering plants (Krizek and Anderson 2013; Weiss et al. 2005). Flower size generally reflects petal size and exhibits remarkable variation among and within species (Guan et al., 2025). Like other organs, a flower's final size is determined by total cell number and cell size, which are influenced by the duration and rate of cell division and cell expansion, respectively (Eloy et al. 2011; Jing et al. 2023; Krizek and Anderson 2013; Rojas et al. 2009). The growth of organs in general and petals in particular occurs in four stages: (1) primordium initiation, (2) cell proliferation, (3) the proliferation-to-expansion transition, and (4) cell expansion (Gonzalez et al. 2012; Hepworth and Lenhard 2014; Jing et al. 2023). These steps are linked to the spatiotemporal expression of genes related to cell division, cell expansion, and cell arrangement (Anastasiou and Lenhard 2007), and are regulated through the intricate coordination of intrinsic growth signals and external environmental cues.

Genetic studies suggest that multiple independent pathways determine flower size by affecting cell proliferation and expansion. Phytohormones, including auxin, cytokinin, ethylene, and jasmonic acid (JA), participate in these pathways (Guan et al., 2025; Breuninger and Lenhard 2010; Krizek and Anderson 2013; Weiss et al. 2005; Ma et al. 2025). For instance, the auxin-responsive transcription factor AUXIN RESPONSE FACTOR 8 (ARF8) acts downstream of the signaling pathway for the plant growth hormone auxin, collaborating with BIG PETAL p (BPEp) to inhibit mitotic activity during the early stages of petal development and to modulate cell expansion at later stages in *Arabidopsis thaliana* (Szécsi et al. 2006; Varaud et al. 2011). Several other genes in the auxin signaling pathway promote petal enlargement by extending the duration of cell division in petals, including *AINTEGUMENTA* (*ANT*) and *AINTEGUMENTA-like 6* (*AIL6*), as well as the C2H2 zinc-finger transcription factor gene *JAGGED* (*JAG*) (Hu et al. 2003; Krizek 2009; Mizukami and Fischer 2000). In rose (*Rosa hybrida*), silencing of *RhARF2* results in larger petals and delayed petal movement (Chen et al. 2023); *RELATED TO AP2.4-Like* (*RhRAP2.4L*) controls cell proliferation and expansion to regulate petal growth (Robil 2024; Wang et al. 2024). The RESPONSE REGULATOR 1 (RhRR1)–SCARECROW-LIKE 28 (RhSCL28) module positively regulates petal size by promoting cell division (Jin et al. 2025). The phytohormone ethylene also affects cell expansion in plant development (Ma et al. 2008; Pei et al. 2013). Silencing the ethylene-responsive transcription factor gene *RhNAC100* in *Rosa hybrida* significantly increases petal size and promotes cell expansion in the petal abaxial subepidermis (Pei et al. 2013). The ethylene-responsive DELLA gene *GIBBERELLIN INSENSITIVE 1* (*RhGAI1*), a direct target gene of ETHYLENE-INSENSITIVE 3 (EIN3), regulates ethylene-mediated cell expansion in rose petals by suppressing the expression of the cellulose synthase gene *RhCesA2* (Luo et al. 2013). Cytokinins are of great importance in plant development, as they control cell division (Zhao et al. 2024). Cytokinin catabolism is catalyzed by cytokinin oxidases/dehydrogenases (CKXs) (Bartrina et al. 2011). In *Arabidopsis*, the *ckx3* *ckx5* double mutant produces larger flowers than the wild type or the two corresponding single mutants due to delayed onset of cellular differentiation and an extended cell division period in the inflorescence and floral meristems (Bartrina et al. 2011). JA orchestrates plant development and stress responses to pathogens and environmental fluctuations, and regulates multiple aspects of plant reproduction, including the flowering transition, floral organ development, anthocyanin biosynthesis, and flower opening (Huang et al., 2025; Yuan and Zhang 2015). JA influences petal development, promoting cell division in early stages along with cytokinin, but inhibiting cell elongation in late stages by activating BPEp, in a bidirectional regulatory model of "promotion followed by inhibition" (Gong et al. 2024; Guan et al. 2022; Guan et al., 2025). Nevertheless, the specific mechanism by which JA signaling modulates flower size through cell expansion remains to be elucidated.

Upon perception by plant cells, JA triggers global transcriptional reprogramming predominantly dictated by the master transcription factor MYC2 (Breeze 2019; Zhai et al. 2020). However, MYC2 function relies on its physical association and functional cooperation with MED25, a critical subunit of the Mediator coactivator complex. MED25 engages in protein–protein interactions with diverse genetic and epigenetic modulators, and orchestrates virtually all MYC2-mediated transcriptional changes (Zhai et al. 2020). In *Arabidopsis*, loss-of-function mutants of *MED25* exhibit enlarged organs, characterized by increased cell size and a modest increase in cell number compared with the wild type. This phenotype arises from longer cell proliferation and cell expansion phases during organ development (Krizek and Anderson 2013; Xu and Li 2011). In addition to its well-characterized role in transcriptional activation, MYC2 has a less-explored role in transcriptional repression, which involves interactions with MED25 and regulators of chromatin state (Çevik et al. 2012; Liu et al. 2019). Notably, transcriptome reprogramming during petal cell growth is also regulated at the level of chromatin remodeling and modification. In particular, histone acetylation has emerged as a major regulator of the petal developmental gene network (Huang et al., 2021). For instance, in *Rosa hybrida*, RhMYB73 recruits the corepressor TOPLESS (RhTPL) and the histone deacetylase RhHDA19 to form a repressive complex (Jing et al. 2023). This complex inhibits the expression of the microRNA locus *RhMIR159* by regulating the acetylation level of histone H3 on lysine 9 (H3K9), thereby leading to a shorter cell division phase and smaller petals (Jing et al. 2023). Similarly, in *Arabidopsis*, HDA6 and HDA9 coordinately regulate silique valve cell elongation by controlling the expression of auxin-related genes in silique tips (Yuan et al. 2019). These results suggest that chromatin modifications and histone deacetylases play critical roles in the development of plant organs, including petals, by regulating cell expansion. In this study, we investigated the effects of JA treatment on rose flower growth and detected elevated transcript levels of *RcMYC2*, which accompanied changes in flower size and cell expansion. Furthermore, silencing *RcMYC2* enhanced late-stage petal cell expansion and resulted in larger flowers. Molecular assays demonstrated that RcMYC2 directly represses *RcEXPA8* by binding to its promoter and recruiting the co-regulator RcMED25 and the histone deacetylase RcHDA8, forming a repressive microenvironment through histone deacetylation that restricts cell expansion. To our knowledge, our study provides the first evidence of an epigenetic regulatory mechanism whereby JA signaling coordinates a transcription factor–Mediator–histone deacetylase complex to fine-tune petal cell expansion.

## Results

### Effects of MeJA treatment or inhibition of JA biosynthesis on floral organ development in roses

**Fig. 1:**
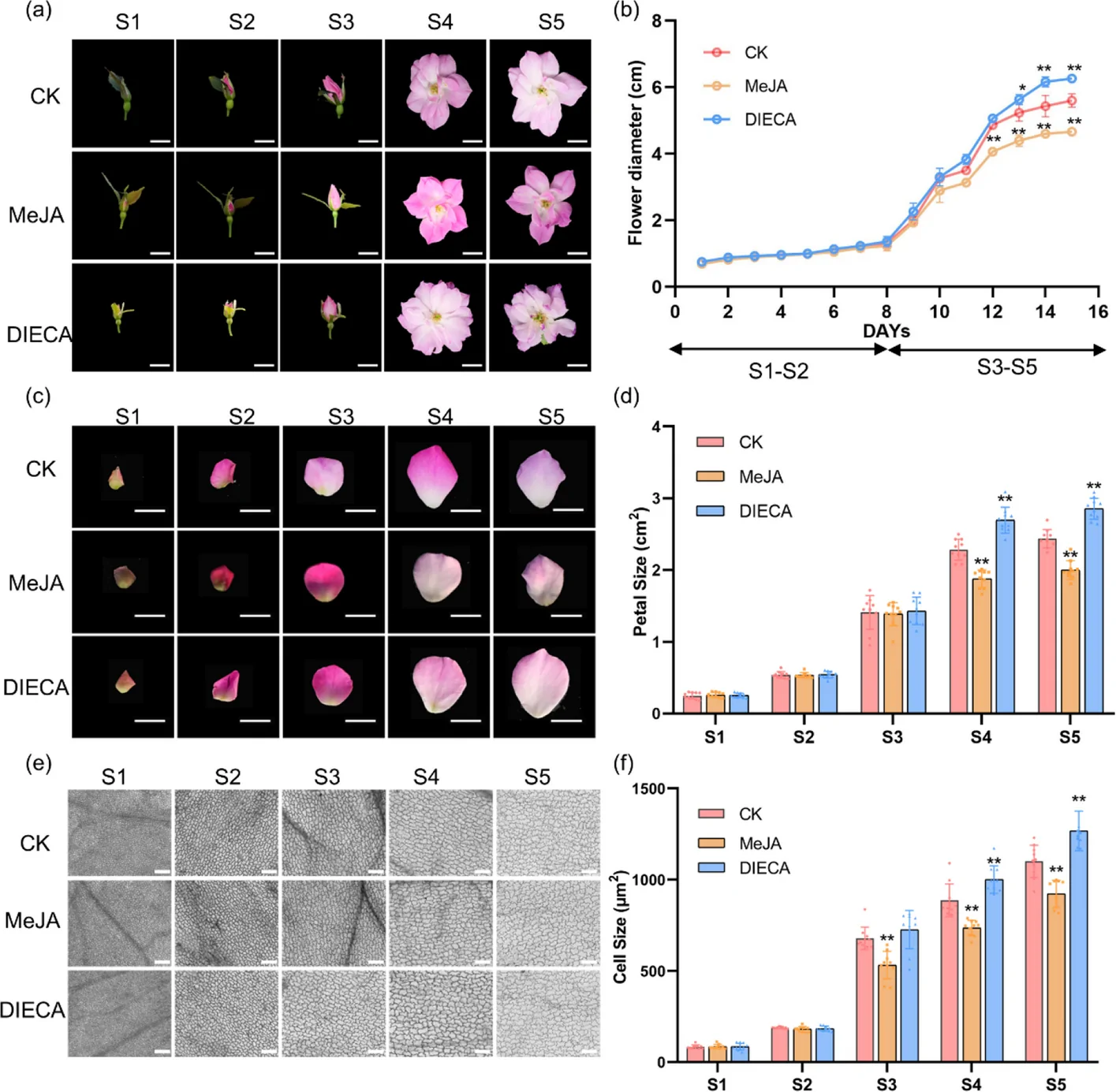
The effects of JA signaling on flower size in Rosa chinensis. **a** Representative photographs showing the different stages of flower development (1–5) of flowers treated with 100 μM MeJA, 1 mM the JA biosynthetic inhibitor DIECA, or deionized water as the control (CK). Stage 1, most sepals are tightly closed; Stage 2, sepals are slightly open with red outer petals; Stage 3, flowers form upright cups without full expansion; Stage 4, flowers are fully open reaching maximum diameter; Stage 5, flowers begin to wilt with curling petal edges. Scale bars, 1 cm. **b** Differences in flower diameter in flower developmental stages 1–5 under MeJA, DIECA, and mock treatments. (*, *P* < 0.05; **, *P* < 0.01; Student’s t-test). **c** Representative photographs of rose flower petals at developmental stages 1–5 under MeJA, DIECA, or mock treatments. Scale bars, 1 cm. **d** Statistical analysis of petal size shown in (**c**). Values are means ± standard deviation (SD) from the petals of at least 10 individual flowers. (* indicates significant differences compared with CK, *P* < 0.05; ** indicates extremely significant differences compared with CK, *P* < 0.01; Student’s t-test). **e** Representative microscopy images of abaxial epidermal cells in the middle region of rose petals across stages 1–5 under MeJA, DIECA, or mock treatments. Scale bars, 50 μm. **f** Statistical analysis of the cell size shown in (**e**). Values are means ± SD from 10 individual flowers. Asterisks indicate significant differences (* indicates significant difference compared with CK, *P* < 0.05; ** indicates extremely significant difference compared with CK, *P* < 0.01; Student’s t-test)

Previous studies have shown that JA regulates floral organ development (Gong et al. 2024; Jing et al. 2023). To investigate the effects of JA signaling on flower growth in more detail, we continuously treated rose flower buds from the initiation stage to stage 1 (when most sepals are tightly closed) (see Supplemental Figure S1), with MeJA, the JA biosynthesis inhibitor sodium diethyldithiocarbamate (DIECA), or distilled water (mock) as a control (see Materials and Methods). We then examined flowers at developmental stages 1–5, as defined in previous studies (Ma et al. 2008; Wang et al., 2024), and measured floral organ parameters, including flower diameter and petal size. No significant differences were detected in flower diameter and petal size across stages 1–2 among the three treatment groups (Fig. 1a–d). However, flowers treated with MeJA had significantly smaller diameters at stage 4 (full bloom) and stage 5 (flowers beginning to wilt with curling petal edges), with an average reduction of ~17% at stage 5 (*n* = 10, *P* < 0.05). Correspondingly, the petals of MeJA-treated flowers were ~18% smaller on average at stages 4 and 5 compared with the control group (stage 4: 1.88 ± 0.12 cm^2^ vs. control: 2.28 ± 0.15 cm^2^; stage 5: 2.01 ± 0.13 cm^2^ vs. control: 2.44 ± 0.13 cm^2^; *n* = 10, *P* < 0.05 for both stages). By contrast, DIECA treatment significantly increased flower diameter and petal size compared with the control; this effect became pronounced at stages 4 and 5. Specifically, under DIECA treatment, petal size was larger by 18% at stage 4 (2.69 ± 0.18 cm^2^) and by 17% at stage 5 (2.86 ± 0.15 cm^2^), with statistical significance (*n* = 10, *P* < 0.05 for both stages). Collectively, these data indicate that MeJA treatment inhibits floral organ development in roses, resulting in smaller flowers.

To investigate the cellular basis of these phenotypic changes, we next measured the size of petal cells. Consistent with the petal growth phenotypes, no significant differences were detected at stages 1–2 in either MeJA or DIECA treatments compared with the control group. Petal cell size began to differ at stage 3 under MeJA treatment, with pronounced differences observed at stages 4 and 5: smaller cells were found in the MeJA treatment group, and larger cells in the DIECA treatment group (Fig. 1e–f). These results indicate that the MeJA-mediated effects on cell expansion primarily occur during late development, specifically from stage 3 to stage 5.

### RcMYC2 negatively regulates rose flower size by inhibiting cell expansion

**Fig. 2:**
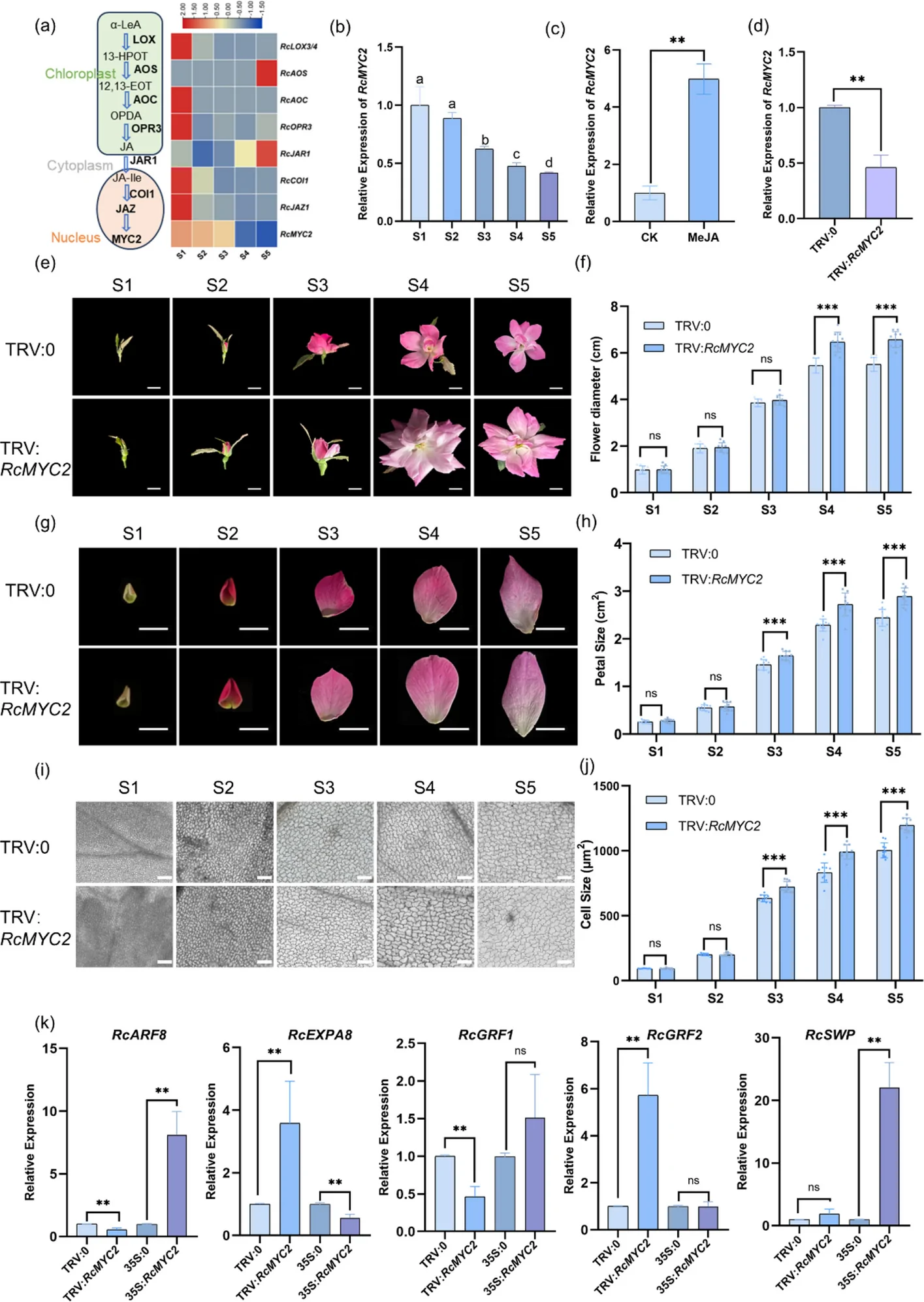
Knockdown of *RcMYC2* leads to larger flowers in *Rosa chinensis*. **a** Heatmap illustrating the expression levels of JA pathway genes across various stages of flower development. Expression values (TPM) are obtained from RNA-seq datasets. **b** Relative expression levels of *RcMYC2* during flower developmental stages 1–5. **c** Relative expression levels of *RcMYC2* in stage 1 of rose flower buds following the same protocol of spraying 100 µM MeJA as described in Fig. 1a. (**, *P* < 0.01; Student’s *t*-test). **d** Relative expression levels of *RcMYC2* in *RcMYC2*-knockdown (TRV:*RcMYC2*) and control (TRV:0) flowers at stage 1. (**, *P* < 0.01; Student’s *t*-test). **e** Representative photographs of flowers at stages 1–5 from TRV:0 and TRV:*RcMYC2* plants. Scale bars, 1 cm. **f** Flower diameter from stages 1–5 shown in (**e**). Values are means ± SD (*n* = 10). (**, *P* < 0.01; ***, *P* < 0.001; Student’s *t*-test). **g** Representative photographs of petals from TRV:0 and TRV:*RcMYC2* plants. Scale bars, 1 cm. **h** Quantification of petal area in TRV:0 and TRV:*RcMYC2* plants. Values are means ± SD (*n* = 10). (**, *P* < 0.01; ***, *P* < 0.001; Student’s *t*-test). **i** Representative microscopy images of petal cells in TRV:0 and TRV:*RcMYC2* plants. Scale bars, 50 μm. **j** Quantification of petal cell area in TRV:0 and TRV:*RcMYC2* plants. Values are means ± SD (*n* = 10). (**, *P* < 0.01; ***, *P* < 0.001; Student’s *t*-test). **k** Relative expression levels of floral organ size genes in 35S:*RcMYC2* and TRV:*RcMYC2* plants. (**, *P* < 0.01; two-tailed Student’s *t*-test)

To clarify the role of JA signaling in regulating rose floral development, we analyzed the expression patterns of key genes in the JA signaling pathway using RNA-seq data (unpublished datasets). As shown in Fig. 2a, the expression levels of most JA pathway genes, particularly *RcCOL1*, *RcJAZ* and *RcMYC2*, gradually declined across stages 1–5 as flower size increased during development. As a core component of the JA signaling pathway, MYC2 is critical to this regulatory process (Breeze 2019). Therefore, we measured the expression of *RcMYC2* by qRT-PCR across stages 1–5 in untreated plants (Fig. 2b) and at stage 1 in plants treated with MeJA (Fig. 2c). The expression of *RcMYC2* gradually decreased during the development of rose flowers and was strongly induced by MeJA treatment, consistent with the RNA-seq data and the phenotypic changes that occurred under MeJA treatment. These results suggest a negative correlation between *RcMYC2* and flower size.

To obtain genetic evidence for the role of *RcMYC2* in regulating rose flower size, virus-induced gene silencing (VIGS) was employed to knock down *RcMYC2* in *Rosa chinensis* ‘Old Blush’ seedlings (Fig. 2d). Notably, knocking down *RcMYC2* (TRV:*RcMYC2*) significantly increased the final flower diameter by approximately 19%, as observed at both stage 4 (6.46 ± 0.43 cm vs control: 5.46 ± 0.32 cm; *n* = 10, *P* < 0.05) and stage 5 (6.57 ± 0.34 cm vs control:5.50 ± 0.30 cm; *n* = 10, *P* < 0.05). This effect was also reflected in significantly larger petal size and cell size from stage 3–5 compared with the TRV:0 control (Fig. 2g–j). Specifically, in *RcMYC2*-knockdown plants (TRV:*RcMYC2*), petals were 12% larger at stage 3 (1.64 ± 0.1 cm^2^ vs control: 1.46 ± 0.11 cm^2^), 19% larger at stage 4 (2.72 ± 0.24 cm^2^ vs control: 2.29 ± 0.13 cm^2^) and 18% larger at stage 5 (2.89 ± 0.18 cm^2^ vs control: 2.44 ± 0.18 cm^2^), with statistical significance (*n* = 10, *P* < 0.05 for stages 3–5) (Fig. 2g, h). Correspondingly, the cells were significantly larger in TRV:*RcMYC2* petals than in the TRV:0 control, with cell areas that were 14% larger at stage 3 (721.17 ± 28.7 μm^2^ vs control: 633.09 ± 26.16 μm^2^), 19% larger at stage 4 (990.76 ± 54.84 μm^2^ vs control: 831.38 ± 74.53 μm^2^), and 19% larger at stage 5 (1195.74 ± 56.63 μm^2^ vs control: 1002.65 ± 58.58 μm^2^) (Fig. 2i, j). Furthermore, we generated *RcMYC2* heterologous overexpression lines in *Arabidopsis*. Preliminary results showed that it significantly reduced floral organ size (Supplemental Figure S2). These results indicate that *RcMYC2* negatively regulates rose floral size by restricting cell expansion, particularly during late development.

To gain deeper insights into how *RcMYC2* regulates flower size at the molecular level, we used RT-qPCR to measure the transcript levels of several key cell expansion-related genes that are involved in flower size regulation (Krizek and Anderson 2013) in *RcMYC2* knockdown and transient overexpression lines. As shown in Fig. 2k, the expression of most tested genes was significantly affected by *RcMYC2* knockdown or overexpression in flowers. Notably, the expansin (*EXP*) gene *RcEXPA8* was highly upregulated in *RcMYC2*-knockdown lines but significantly downregulated in overexpression lines, suggesting that RcMYC2 may directly regulate expansin (*EXPA*) genes and thereby influence cell expansion. Collectively, these results indicate that *RcMYC2* negatively regulates rose flower size by inhibiting cell expansion.

### RcMYC2 directly binds to the *RcEXPA8* promoter and represses its transcription

**Fig. 3:**
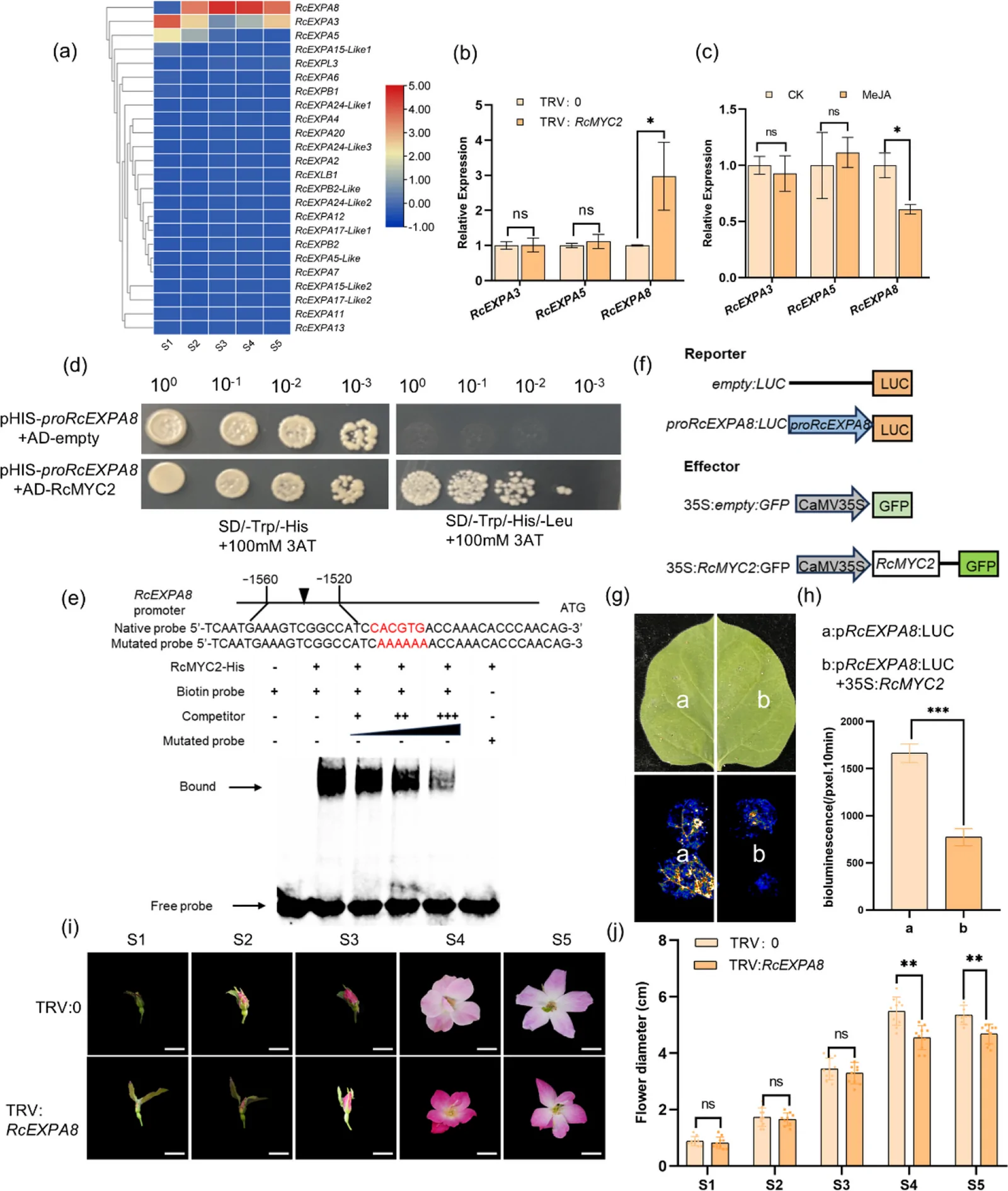
RcMYC2 binds to the *RcEXPA8* promoter and suppresses its transcription. **a** Heatmap representation of expression levels for *RcEXP* genes across flower stages 1–5 in *Rosa chinensis*. The values are TPM. Row-wise normalization. **b** Relative expression levels of *RcEXPA3*, *RcEXPA5*, and *RcEXPA8* in rose petals were determined by RT-qPCR following treatment with distilled water (mock) or 100 µM MeJA, with values presented as means ± SD (*n* = 3). (*, *P* < 0.05; **, *P* < 0.01, Student’s *t*-test). **c** Relative expression levels of *RcEXPA3*, *RcEXPA5*, and *RcEXPA8* in the petals of TRV:0 and TRV:*RcMYC2* plants, as determined by RT-qPCR. Values are means ± SD (*n* = 3). (*, *P* < 0.05; **, *P* < 0.01, Student’s *t*-test). **d** Yeast one-hybrid assay showing that RcMYC2 binds to the *RcEXPA8* promoter. SD, synthetic defined medium. AD-empty, pGADT7 vector; AD-*RcMYC2*, RcMYC2 fused to the GAL4 activation domain. **e** Electrophoretic mobility shift assay showing the binding of recombinant purified RcMYC2-His in vitro to a DNA probe containing the G-box element of the *RcEXPA8* promoter. **f** Diagram of the effector and reporter constructs used in the LUC reporter assay. **g** RcMYC2 inhibits the promoter activity of RcEXPA8. The upper image shows the brightfield and the lower image shows the LUC activity. **h** Quantification of firefly luciferase activity for each experimental combination. Values are means ± SD (*n* = 3). (***, *P* < 0.001, Student’s *t*-test). **i** Representative photographs of flowers from stages 1–5 of TRV:0 and TRV:*RcEXPA8* plants. Scale bars, 1 cm. **j** Flower diameter from stages 1–5 shown in (**i**). (**, *P* < 0.01; Student’s *t*-test)

Expansins are a class of cell wall proteins that loosen the cell wall, thereby promoting cell elongation and expansion and determining cell size (Cosgrove 2005). Based on our results, we speculated that RcMYC2 may participate in rose petal cell expansion by regulating the expression of cell expansion-related genes, particularly the expansin (*EXP*) gene *RcEXPA8*. Therefore, we further identified all *RcEXP* genes in the rose genome and analyzed their expression profiles during stages 1–5 of rose flower development. As shown in Supplemental Figure S3 and Fig. 3a, 24 *RcEXP* genes were identified in the rose genome and among them only three *RcEXPA* genes, *RcEXPA3*, *RcEXPA5*, *and RcEXPA8*, were differentially expressed during petal developmental stages 1–5. Of these genes, only *RcEXPA8* (RchiOBHm_Chr5g0072761) was significantly downregulated in the petals of MeJA-treated flowers and upregulated in the petals of *RcMYC2*-knockdown plants (Fig. 3b, c).

Then, we asked whether RcMYC2 directly binds to the *RcEXPA8* promoter to repress its expression by conducting yeast one-hybrid (Y1H) and luciferase (*LUC*) reporter assays. Previously, MYC2 transcription factor has been reported to specifically bind to the G-box (CACGTG) motif in the promoters of their downstream target genes (Yadav et al. 2005). Using the JASPAR CORE database (https://jaspar.genereg.net/), two G-box (CACGTG) motifs were predicted in the *RcEXPA8* promoter. Y1H assays showed that RcMYC2 specifically binds the G-box in the P1 region of the *RcEXPA8* promoter, but does not bind the other putative G-box motif (Fig. 3d). This binding was further validated by electrophoretic mobility shift assays (EMSAs): biotin-labeled probes corresponding to the P1 region bound to recombinant His-tagged RcMYC2, and the binding intensity decreased as increasing amounts of unlabeled competitor probes were added (Fig. 3e). In dual-luciferase assays, co-infiltration of *35S:RcMYC2* with *proRcEXPA8:LUC* into *N. benthamiana* leaves resulted in significantly lower luciferase activity than that derived from *proRcEXPA8:LUC* alone, confirming that RcMYC2 acts as a transcriptional repressor of *RcEXPA8* transcription (Fig. 3f–h).

To characterize the function of *RcEXPA8* in flower size regulation, we knocked down its expression using VIGS and compared flower development between TRV:0 control plants and *RcEXPA8*-knockdown plants (Fig. 3i). During stages 1–3, no significant difference in flower size was observed between the two groups. During stages 4–5, however, the flowers of *RcEXPA8* knockdown plants were significantly smaller than those of the TRV:0 control (Fig. 3j). Specifically, the final flower diameter of *RcEXPA8* knockdown plants was 4.55 ± 0.43 cm, compared with 5.49 ± 0.50 cm in the TRV:0 control plants. These results indicate that *RcEXPA8* functions as a positive regulator of flower size in roses. The smaller flower phenotype observed in *RcEXPA8* knockdown plants likely reflects impaired petal cell expansion, as expansins (EXPs) promote cell wall loosening and tissue elongation (Cosgrove 2005).

### RcMYC2 requires the mediator subunit *RcMED25* to regulate flower size in rose

**Fig. 4:**
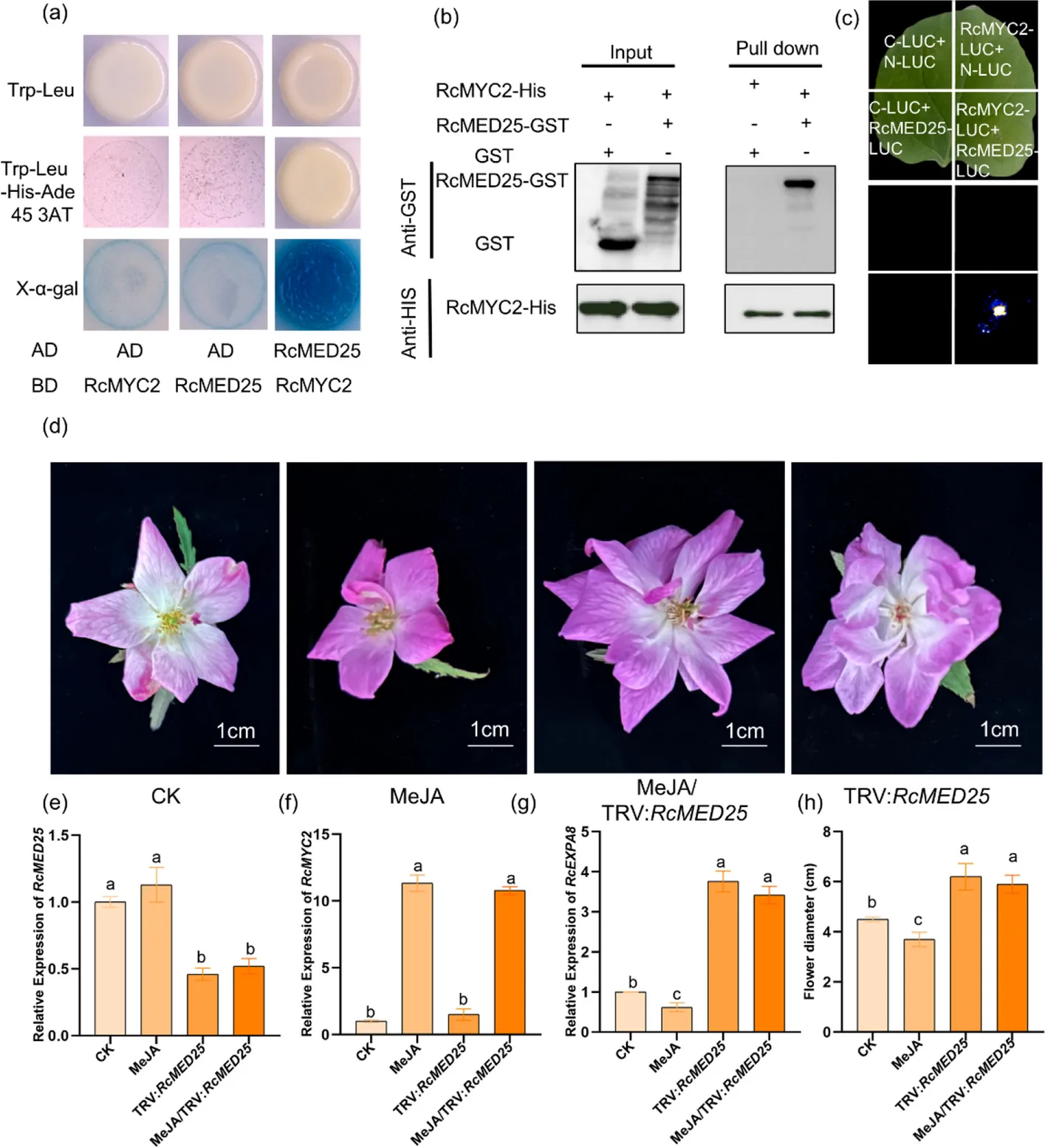
RcMYC2 interacts with RcMED25 and requires RcMED25 function to regulate rose flower size. **a** Yeast two-hybrid assay demonstrating the interaction between RcMYC2 and RcMED25. Self-activation by RcMYC2 was inhibited by the addition of 45 mM 3-AT. AD, GAL4 activation domain; BD, GAL4 DNA-binding domain. **b** In vitro pull-down assay confirming the physical interaction between recombinant purified RcMYC2-His and RcMED25-GST. **c** Bimolecular luciferase complementation assay in *N. benthamiana* leaves verifying the RcMYC2–RcMED25 interaction in vivo. C-LUC, C-terminal half of firefly luciferase (LUC); N-LUC, N-terminal half of LUC. **d** Representative photographs of flowers from plants under different treatment conditions: mock treatment, MeJA treatment, MeJA treatment combined with *RcMED25*-knockdown (MeJA/TRV:*RcMED25*), and *RcMED25*-knockdown (TRV:*RcMED25*). Scale bars, 1 cm. **e**–**g** Relative expression levels of *RcMED25* (**e**), *RcMYC2* (**f**), and *RcEXPA8* (**g**) in the flowers of mock-treated or MeJA-treated wild-type and TRV:*RcMED25* plants, as determined by RT-qPCR. Values are means ± SD (*n* = 3). Different lowercase letters indicate significant differences, as determined by one-way ANOVA followed by Tukey’s HSD test (*P* < 0.05). **h** Flower diameter across treatment groups. Values are means ± SD (*n* = 3). Different lowercase letters indicate significant differences, as determined by one-way ANOVA with Tukey’s HSD test (*P* < 0.05)

The Mediator complex functions as a molecular bridge that connects transcription factors to the transcription machinery, thereby regulating transcriptional activity (Malik and Roeder 2010). Consequently, identifying specific Mediator complex subunits involved in transducing regulatory signals from transcription factors to downstream target genes is of considerable research significance. In *Arabidopsis*, MED25 functions as a subunit of the Mediator complex and negatively regulates floral organ size (Xu and Li 2011). Additionally, MED25 interacts with MYC2 to either activate or repress the expression of downstream target genes. However, whether RcMYC2 regulates floral organ size in roses via the JA pathway in a RcMED25-dependent manner remains unclear. Thus, to address this question, we first performed targeted yeast two-hybrid (Y2H), split-luciferase (LUC) complementation, and pull-down assays to investigate the interactions between RcMYC2 and RcMED25. As shown in Fig. 4a–c, all three assays confirmed their interaction.

To confirm the role of RcMED25 in regulating rose flower size, we generated *RcMED25*-knockdown lines (TRV:*RcMED25*) using VIGS. Quantitative analysis confirmed that *RcMED25* transcript levels were significantly reduced in flowers of TRV:*RcMED25* plants compared with the TRV:0 control (Supplemental Figure S4a). Phenotypically, the knockdown of *RcMED25* resulted in a significant increase of ~13% in final flower diameter and ~22% in final petal size. These results indicate that RcMED25 functions as a negative regulator of flower size in rose (Supplemental Figure S4b, c).

To investigate whether the process by which RcMYC2 regulates flower size in response to JA signaling requires RcMED25, we first examined the expression response of *RcMED25* to MeJA treatment. As we expected, no significant changes in relative *RcMED25* transcript levels were detected in rose flowers under MeJA treatment compared with the mock control, indicating that JA does not directly stimulate *RcMED25* expression (Fig. 4e). This finding suggests that RcMED25 acts as a cofactor for RcMYC2 in the JA pathway.

To further investigate the interplay and functional dependency between RcMYC2 and RcMED25 in the regulation of rose flower size, we induced higher transcript levels of *RcMYC2* via MeJA treatment in *RcMED25*-knockdown lines. As shown in Fig. 4d–h, MeJA-induced upregulation of *RcMYC2* failed to reduce flower size in *RcMED25*-knockdown lines compared with MeJA-treated wild-type controls, whereas the flower size remained comparable to that in *RcMED25*-knockdown lines without MeJA treatment. Correspondingly, the expression level of *RcEXPA8*, a positive regulator of flower size, was not recovered under the MeJA treatment in *RcMED25*-knockdown lines (Fig. 4g). These results indicate that RcMYC2-mediated JA signaling in rose flower size regulation requires RcMED25.

### RcMED25 recruits RcHDA8 to demethylate histones at the *RcEXPA8* promoter

**Fig. 5:**
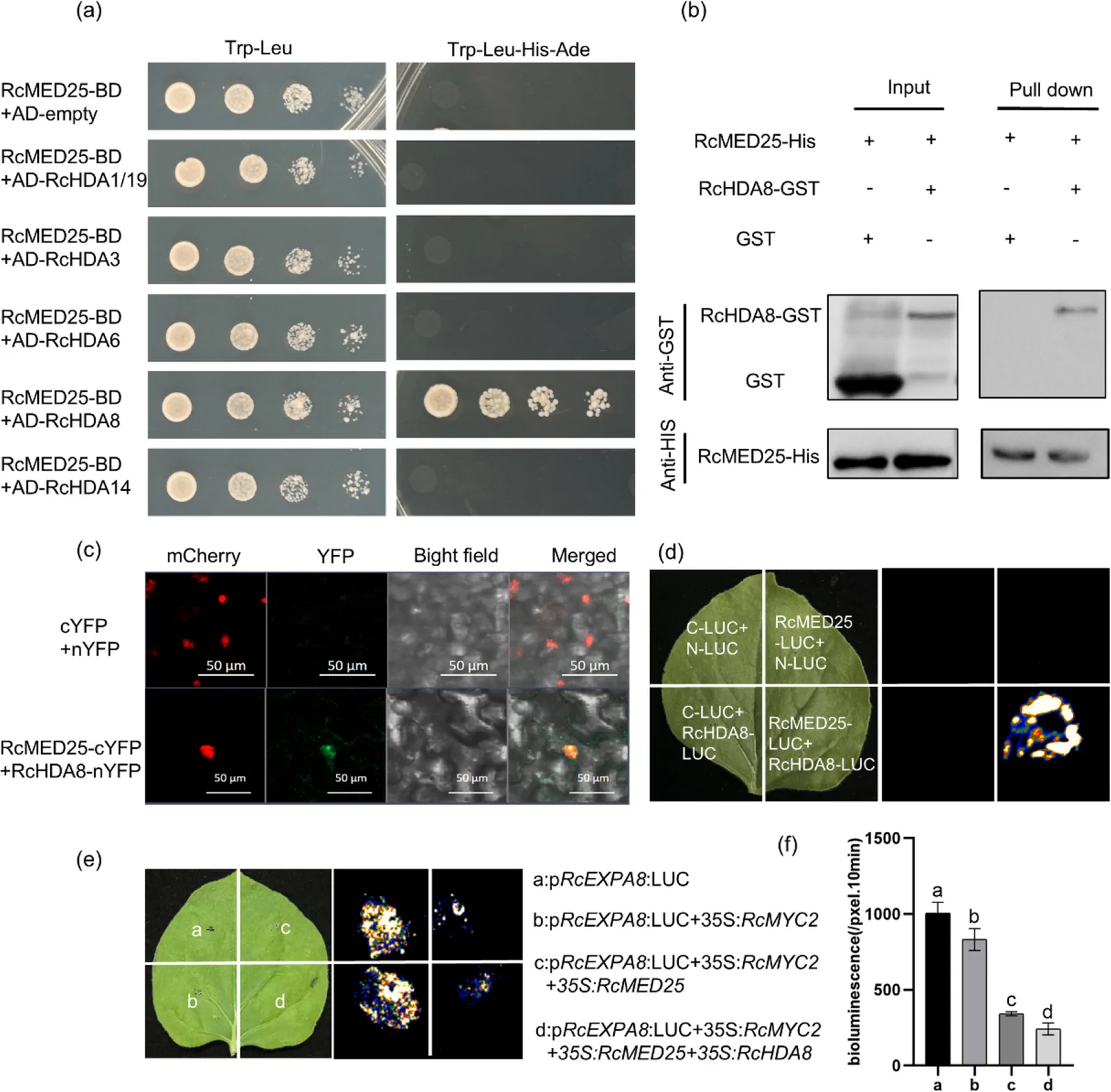
RcMED25 and RcHDA8 physically interact in vitro and in vivo. **a** Yeast two-hybrid assay demonstrating the interaction between RcMED25 and RcHDA8. **b** In vitro pull-down assay demonstrating the direct physical interaction between recombinant purified RcMED25-His and RcHDA8-GST. **c** Bimolecular fluorescence complementation (BiFC) assay revealing the interaction between RcMED25 and RcHDA8. *Nicotiana benthamiana* leaves were co-infiltrated with the *RcMED25-nYFP* and *RcHDA8-cYFP* constructs. YFP fluorescence was detected by confocal microscopy 3 days post-infiltration. Scale bars, 50 µm. **d** Luciferase (LUC) assays confirming the interaction between RcMED25 and RcHDA8. *N. benthamiana* leaves were co-infiltrated with constructs encoding RcMED25-CLUC and RcHDA8-NLUC or with the corresponding empty vector controls. LUC activity was detected 3 days after infiltration. **e** Dual-luciferase reporter assay showing that the presence of RcMED25 and RcHDA8 enhances the RcMYC2-mediated transcriptional repression of *RcEXPA8* in *N. benthamiana* leaves. Left, brightfield image of a representative infiltrated *N. benthamiana* leaf; right, luciferase activity. **f** Quantification of LUC activity for each experimental group shown in (**e**). Different lowercase letters indicate significant differences, as determined by one-way ANOVA followed by Tukey’s multiple comparisons test (*P* < 0.05)

Chromatin remodeling and modifications regulate petal cell growth (Guan et al., 2025), with histone acetylation serving as a key regulator of petal development (Huang et al., 2021; Jing et al. 2023). Its status is tightly controlled by antagonistic HATs and HDACs, with HDACs removing acetyl groups to repress transcription (Charron et al. 2009; Guo et al. 2023). Previous studies suggested that the MED25–MYC2 complex may recruit unknown repressive cofactors (Zhai et al. 2020); our study further suggests that this complex mediates promoter looping at *RcEXPA8* to form a repressive microenvironment during JA signaling. An intriguing possibility is that HDACs might be involved in forming a repressive complex with MED25–MYC2, thereby contributing to the inhibition of *RcEXPA8* expression. To test this, we first identified 14 histone deacetylase (HDAC) genes in the rose genome (Supplemental Figure S5a). We then systematically examined the pairwise interactions between RcMYC2 or RcMED25 and each of these 14 putative RcHDACs using split-luciferase (LUC) assays. As shown in Supplemental Figure S5b, none of the RcHDACs interacted with RcMYC2, but five of them did interact with RcMED25. To validate these findings, we further performed yeast two-hybrid (Y2H) assays, which confirmed that only RcHDA8 (RchiOBHm_Chr5g0048241) directly interacts with RcMED25 (Fig. 5a).

To further validate the interaction between RcMED25 and RcHDA8, we also performed in vitro pull-down assays. Recombinant purified RcMED25-His was incubated with either glutathione S-transferase (GST) alone or with RcHDA8-GST, followed by pull-down using His resin. Finally, only RcHDA8-GST was detected in the protein complex pulled down by RcMED25-His (Fig. 5b). In a bimolecular fluorescence complementation (BiFC) assay, reconstitution of yellow fluorescent protein (YFP) signal was exclusively observed in the nuclei of *Nicotiana benthamiana* leaf cells co-expressing RcMED25-cYFP and RcHDA8-nYFP (Fig. 5c), confirming their physical interaction. Finally, transactivation assays revealed that the presence of both RcMED25 and RcHDA8 significantly enhanced the RcMYC2-mediated transcriptional repression of *RcEXPA8* (Fig. 5e, f). Collectively, these results support the functional relevance of the RcMED25–RcHDA8 interaction in regulating *RcEXPA8* expression.

### RcHDA8 regulates histone acetylation levels at the *RcEXPA8* promoter and modulates flower size

**Fig. 6:**
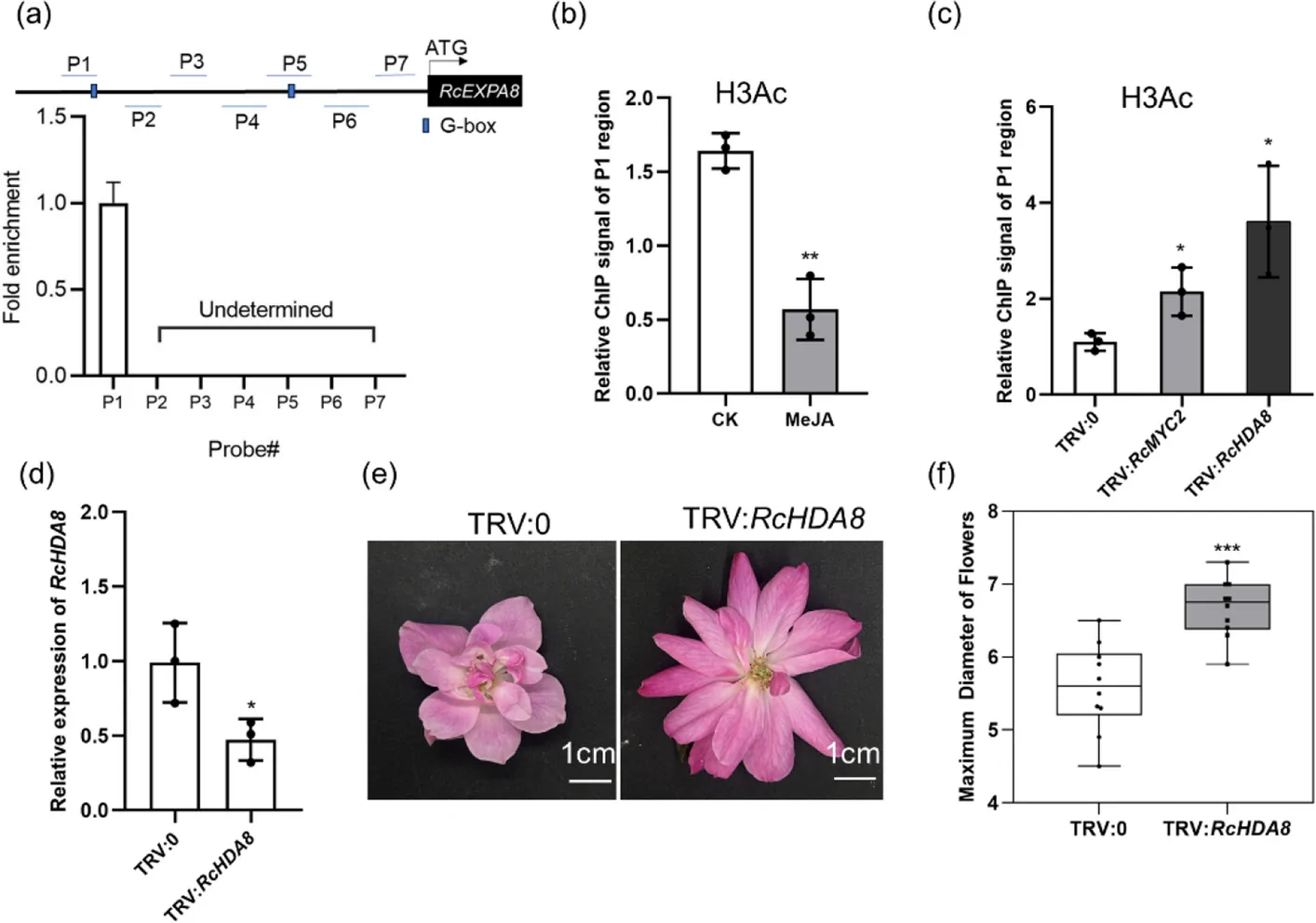
RcHDA8 regulates histone acetylation levels at the *RcEXPA8* promoter and modulates flower size. **a** Diagram of the RcMYC2 recognition sites (top) and relative H3K9/K14Ac levels in the *RcEXPA8* promoter (bottom). Values are means ± SD from three biological replicates (*n* = 3; *, *P* < 0.05; **, *P* < 0.01). Blue boxes indicate G-box *cis*-elements. P1–P7 indicate the PCR amplicons that were amplified during ChIP-qPCR. **b** and **c** Relative H3K9/K14Ac levels along the *RcEXPA8* promoter in the flowers of rose plants treated with JA (**b**) and in *RcMYC2*-knockdown or *RcHDA8*-knockdown plants (**c**). H3Ac enrichment at the P1 region of the *RcEXPA8* promoter was measured using ChIP-qPCR. (*, *P* < 0.05; **, *P* < 0.01, Student’s *t*-test). **d** RT-qPCR analysis of *RcHDA8* expression levels in the petals of TRV:0 and *RcHDA8*-knockdown plants. Values are means ± from three biological replicates (*n* = 3). (**, *P* < 0.01, Student’s *t*-test). *RcUBI2* was used as an internal reference. **e**, **f** Knockdown of *RcHDA8* leads to larger flowers. Values are means ± SD (*n* = 8). Scale bars, 1 cm. Representative photographs (**e**) and diameters of flowers (**f**) from TRV:0 and *RcHDA8*-knockdown plants. Fully opened flowers were measured. The box plots show the median, interquartile range (box edges), whiskers (SD, *n* = 8), and individual data points (dots). (***, *P* < 0.001, Student’s *t*-test)

Previous studies have demonstrated that HDACs regulate histone H3K9 and H3K14 acetylation levels in plants (Luo et al. 2012). To investigate whether the RcMED25–RcHDA8 complex modulates histone acetylation at the *RcEXPA8* promoter, we performed chromatin immunoprecipitation followed by quantitative PCR (ChIP-qPCR) to measure H3 acetylation levels across the *RcEXPA8* promoter in rose petals. Our analysis revealed that H3K9 and H3K14 acetylation levels were relatively high specifically in regions adjacent to the RcMYC2 binding sites (designated as P1, spanning −1798 bp to −1438 bp; Supplementary Table S1) within the *RcEXPA8* promoter (Fig. 6a). Notably, following MeJA treatment, we detected significantly reduced H3 acetylation levels at the *RcEXPA8* promoter, consistent with the hypothesis that JA signaling promotes a repressive chromatin state at this locus.

Furthermore, knockdown of *RcHDA8* or *RcMYC2* resulted in significantly elevated levels of H3K9 and H3K14 acetylation within the P1 region (Fig. 6b, c). These findings strongly suggest that the RcMYC2–RcMED25 complex recruits RcHDA8 to the *RcEXPA8* promoter, where it modulates histone acetylation status. To further investigate the functional role of RcHDA8 in petal development, we employed VIGS to knock down *RcHDA8* expression. RT-qPCR analysis confirmed a 61% reduction in *RcHDA8* transcript levels in flowers of *RcHDA8*-knockdown plants compared with TRV:0 control plants (Fig. 6d). Phenotypically, *RcHDA8* knockdown led to a significant 22% decrease in flower diameter relative to controls (*P* < 0.001; Fig. 6e, f). These results collectively demonstrate that RcHDA8-mediated histone deacetylation plays a critical role in modulating flower size in roses.

**Fig. 7:**
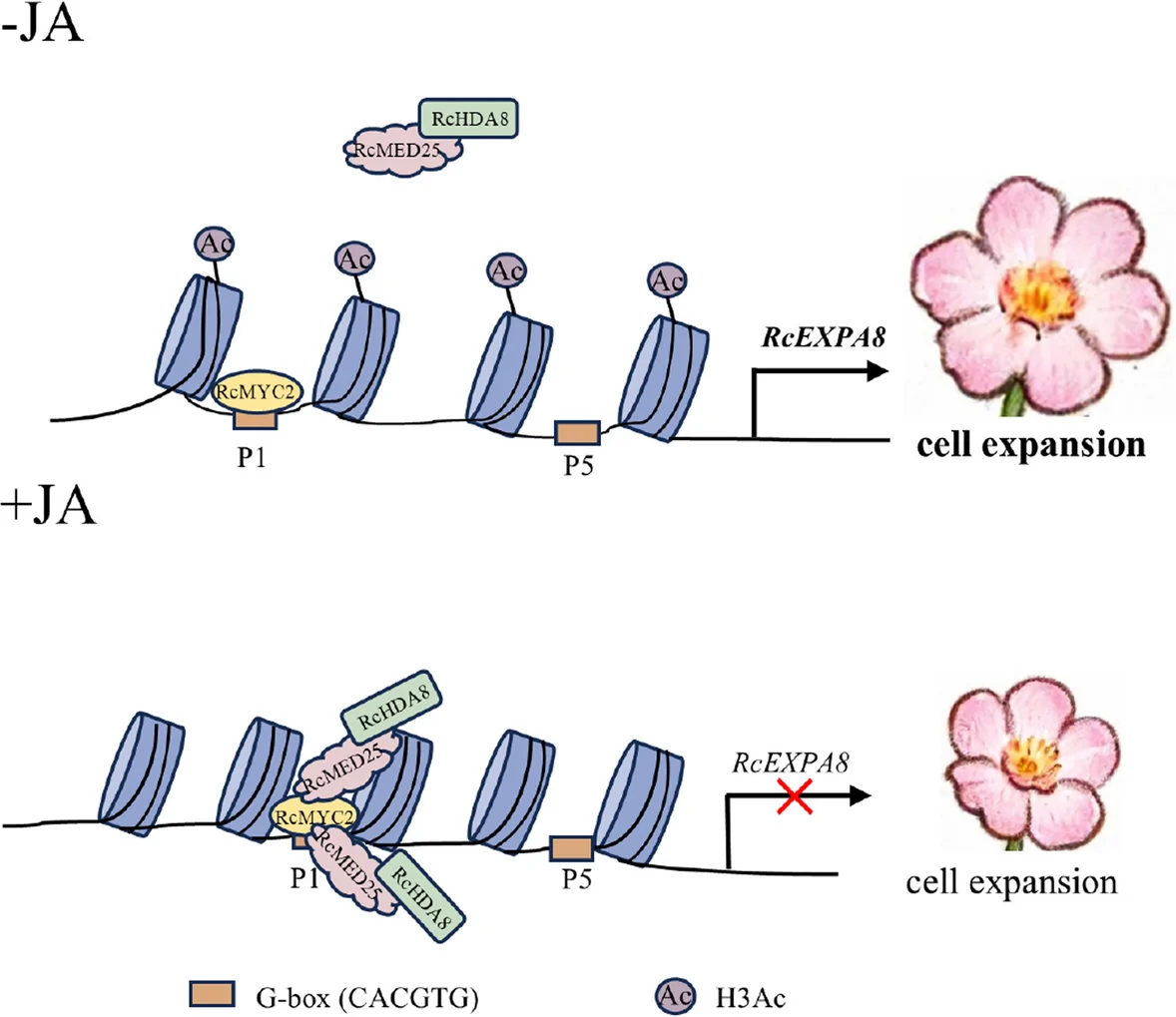
A proposed model describing the function of the RcMYC2–RcMED25–RcHDA8 complex mediating jasmonic acid signaling to regulate flower size via histone deacetylation in *Rosa chinensis*

In this paper, our results collectively establish a model for the role of the RcMYC2–RcMED25–RcHDA8 repressive complex in flower size determination: upon JA signaling, RcMYC2 recruits RcHDA8 to the *RcEXPA8* promoter through RcMED25, forming a transcriptionally repressive microenvironment via histone deacetylation, which ultimately restricts petal cell expansion. This mechanism provides a three-tier regulatory framework involving a transcription factor, a Mediator subunit, and an epigenetic modifier that controls JA-mediated rose flower size (Fig. 7).

## Discussions

Ensuring optimal flower size is critical for plant reproductive fitness and environmental adaptation (Krizek and Anderson 2013; Weiss et al. 2005). JA plays an important role in regulating flower size (Gong et al. 2024; Guan et al., 2025; Jiang et al. 2022). However, JA-mediated control of petal size involves different mechanisms in different species. For example, in *Gerbera hybrida*, JA inhibits ray petal elongation by inducing *GhBPE* expression (Jiang et al. 2022), whereas in *Chrysanthemum morifolium*, the JA signaling repressor CmJAZ1-like restricts cell expansion by suppressing *CmBPE2* transcription (Guan et al. 2022; Guan et al., 2025). Similarly, JA has been implicated in governing late-stage petal growth in *Arabidopsis thaliana* (Brioudes et al. 2009). These results indicate that JA plays a dual role in controlling petal size by affecting both cell proliferation and expansion (Guan et al., 2025). In our study, we continuously treated rose (*Rosa chinensis*) flower buds from initiation stage to stage 1 with MeJA or the JA inhibitor sodium DIECA and systematically measured flower diameter, petal size, and cell size during flower development. The final flower diameter was significantly altered, with a 17% reduction under MeJA treatment and a 15% increase under DIECA treatment during late flower development. Differences emerged at stage 3 and became particularly pronounced at stages 4–5 (Fig. 1a–b). These changes were also reflected in petal size and cell size (Fig. 1c–f), indicating that JA negatively regulates rose flower size by affecting cell expansion during late developmental stages, which is consistent with previous results in *Arabidopsis* (Brioudes et al. 2009). In addition, based on the preliminary JA concentration gradient experiments performed in this study, our results revealed a concentration-dependent effect of JA on the regulation of rose flower size, which represents an important direction for future investigation. Future studies may systematically examine the effects of different JA concentrations on petal development, cell expansion, and associated regulatory pathways, thereby further refining the molecular network underlying JA-mediated control of rose flower size.

As a core component of the JA signaling pathway, MYC2 has long been recognized as a transcriptional activator of defense and secondary metabolism genes in *Arabidopsis* (Luo et al. 2023; Song et al. 2022). However, emerging evidence reveals its role as a repressor in developmental processes, such as inhibiting *FLOWERING LOCUS T* (*FT*) expression to delay flowering (Freytes et al. 2021; Wang et al. 2017) or interacting with FLOWERING LOCUS C to modulate photomorphogenesis (Chakraborty et al. 2025). In rose, MeJA treatment induces the expression of *RhMYC2* and negatively controls petal size by integrating cytokinin and JA signaling (Gong et al. 2024). However, the specific mechanism by which JA signaling modulates flower size via cell expansion remains to be elucidated. Our results in this study showed that the expression of *RcMYC2* negatively correlated with flower diameter during flower development. RcMYC2 directly represses *RcEXPA8,* which encodes a key expansin that promotes cell wall loosening and cell expansion (Cosgrove 2000), by binding to its G-box motif (CACGTG) in the proximal promoter (Fig. 3d–h). This repression is physiologically relevant: silencing *RcMYC2* elevates *RcEXPA8* expression, increases petal cell size by ~19% at stage 5, and enlarges flower diameter by ~19% (Fig. 2d–j), phenocopying the effects of inhibition of JA biosynthesis (DIECA treatment). Notably, this repressive role of *RcMYC2* is stage-specific, primarily impacting late petal development (stages 3–5). This aligns with the temporal duality of JA signaling in rose petals: JA promotes cell division in early stages (stages 0–2) via RhMYC2-mediated activation of cytokinin biosynthesis (Gong et al. 2024), but inhibits cell expansion in late stages through RcMYC2-dependent repression of *RcEXPA8*. Such stage-specificity mirrors the role of JA in chrysanthemum, where it activates BPEp to inhibit late petal elongation (Guan et al. 2022; Guan et al., 2025), suggesting a conserved function of JA in balancing growth. Compared to *Arabidopsis* MYC2, which represses flowering via *FT* (Freytes et al. 2021; Wang et al. 2017), RcMYC2’s targeting of *RcEXPA8* highlights how MYC2 orthologs have evolved distinct downstream targets to regulate lineage-specific traits (e.g., flower size in roses vs flowering time in *Arabidopsis*).

The Mediator subunit MED25 is traditionally viewed as a transcriptional co-activator, bridging transcription factors (e.g., MYC2) to RNA polymerase II in *Arabidopsis* (Chen et al. 2012; Zhai et al. 2020). Our study challenges this paradigm by demonstrating that RcMED25 functions as a scaffold for a repressive complex, interacting with both RcMYC2 and the histone deacetylase RcHDA8 to repress *RcEXPA8* (Figures. 4, 5). This finding aligns with recent reports that *Arabidopsis* MED25 associates with HDA19 to repress light-responsive genes via PIF1/PIF3 (Guo et al. 2023), indicating a conserved role for MED25 in recruiting epigenetic modifiers. However, our work is the first to link this activity to JA-mediated organ size control, expanding MED25’s functional repertoire beyond stress responses to include developmental regulation. Mechanistically, RcMED25 is indispensable for JA signaling in rose petals: MeJA fails to reduce flower size or repress RcEXPA8 in RcMED25-knockdown plants (Fig. 4d–h), even with elevated RcMYC2 levels. This dependency mirrors *Arabidopsis*, where MED25 is required for MYC2-mediated activation of defense genes (Wang et al. 2019), but with a critical twist: in roses, MED25 enables repression by recruiting HDACs rather than activating transcription. This dual role—activating defense genes under stress, but repressing growth genes during development—positions MED25 as a molecular switch that tailors MYC2’s output to physiological context, a concept with broad implications for understanding Mediator function in plants.

Histone acetylation is a well-established regulator of plant organ size, with HDACs often acting as growth repressors by compacting chromatin at growth-promoting genes (Berger 2002). In roses, RhHDA19 is recruited by RhMYB73 to repress *RhMIR159*, limiting cell division and petal size (Jing et al. 2023). Our study extends this paradigm by identifying RcHDA8 as a key effector of JA-mediated cell expansion repression, reducing H3K9 and H3K14 acetylation at the *RcEXPA8* promoter (Fig. 6a–c). This mechanism is conserved: *Arabidopsis* HDA6 and HDA9 repress auxin-responsive genes to control silique valve cell elongation (Yuan et al. 2019), and rice MED25 recruits HDACs to silence auxin signaling (Shapulatov et al. 2023; Suzuki et al. 2022), highlighting HDACs as universal modulators of growth-related gene expression. Notably, RcHDA8 recruitment is JA-dependent and requires the RcMYC2–RcMED25 complex, ensuring precise temporal regulation: under basal conditions, *RcEXPA8* is active (high H3 acetylation) to promote cell expansion; upon JA signaling, RcHDA8 is recruited, deacetylates histones, and represses *RcEXPA8*, restricting further growth. This epigenetic switch likely prevents excessive petal expansion under stress, balancing defense and reproduction—a trade-off central to plant fitness (Huot et al. 2014).

In this study, we unveil a novel regulatory pathway in which the RcMYC2–RcMED25–RcHDA8 complex mediates JA signaling to repress the expansin gene *RcEXPA8* via histone deacetylation, thereby restricting late-stage petal cell expansion. This work advances three key concepts: (1) MYC2 acts as a context-dependent repressor in development, (2) MED25 bridges transcription factors to epigenetic modifiers for repression, and (3) histone deacetylation fine-tunes cell expansion in horticultural crops. By integrating molecular insights with agronomic relevance, this study not only enriches our understanding of JA signaling but also provides tools to engineer flower size in roses and other ornamental plants.

## Materials and methods

### Plant materials and growth conditions

In this study, tissue-cultured seedlings of the ancient Chinese rose 'Old Blush' (*Rosa chinensis* 'Old Blush') with uniform growth status were used as experimental materials. Adventitious buds grown under axenic conditions were placed onto subculture medium (Murashige and Skoog [MS] salts +1.0 mg·L^−1^ 6-BA +0.1 mg·L^−1^ NAA +30 g·L^−1^ sucrose +7.5 g·L^−1^ agar, pH 5.8–6.0) for 20 days of proliferation culture before being transferred to rooting medium (half-strength MS salts +0.1 mg·L^−1^ NAA +30 g·L^−1^ sucrose +7.5 g·L^−1^ agar, pH 5.8–6.0) to promote root development. When root length reached 2–4 cm, the seedlings were transplanted into a nutrient substrate with a vermiculite:peat:perlite ratio of 5:4:1 (w/w/w) and cultivated at 25 °C, 70–80% humidity, under a 16-h light/8-h dark photoperiod.

For JA treatment, we continuously treated the rose flower buds from initiation stage to stage 1 (most sepals are tightly closed), with 100 µM methyl jasmonate (MeJA), 1 mM sodium diethyldithiocarbamate (DIECA), or distilled water (mock) as a control. Flower phenotypes were recorded after blooming, and petal samples were collected from the outermost whorl of the flowers.

### Measurements of petal cell size

Abaxial epidermis (AbsE) cells were observed following the method following the method of Ma et al. (2008). Petal disks (0.5 cm in diameter) were excised from the region three-quarters of the way down the petal apex, fixed overnight in formaldehyde-acetic acid solution (3.7% formaldehyde, 5% glacial acetic acid, 50% ethanol), and dehydrated in absolute ethanol to remove the fixative. Cells were visualized via inverted fluorescence microscopy (OLYMPUS IX73; OLYMPUS, Tokyo, Japan). AbsE cell area was measured using ImageJ software (National Institutes of Health, USA), and cell counts were performed per field of view.

### Reverse-transcription quantitative PCR (RT-qPCR)

Total RNA was extracted from the flowers of rose cuttings using a FastPure Plant Total RNA Isolation Kit (Vazyme, Nanjing, China) and then reverse-transcribed into first-strand cDNA using One-Step gDNA Removal and cDNA Synthesis SuperMix (Transgen Biotech, Beijing, China) following the manufacturers’ instructions. qPCR primers for *RcMYC2* and the reference gene *RcUBI2* were designed using the GenScript Real-Time PCR Primer Design Tool (https://www.genscript.com/tools/real-time-pcr-taqman-primer-design-tool) and synthesized by Qingke Biotechnology Co., Ltd. qPCR was performed using M5 HiPer Real-Time PCR SuperMix (MF013, TransGen Biotech) on a StepOne Plus Real-Time PCR System. Relative expression levels of target genes were calculated using the 2^*−*ΔΔCt^ method, with *RcUBI2* as the internal reference. The primers used in this study are listed in Supplementary Table S1.

### Virus-Induced Gene Silencing (VIGS)

Rose seedlings with target gene knockdown were generated using a VIGS system, following the previously described protocol (Chen et al., 2023). *Agrobacterium* (GV3101) cell suspensions containing the constructs TRV:*RcMYC2*, TRV:*RcMED25*, or TRV:*RcEXPA8* were mixed with an *Agrobacterium* cell suspension harboring pTRV1 in a 1:1 (v/v) ratio and adjusted to an optical density at 600 nm of 0.6. Bacterial cells were harvested by centrifugation at 2200 *g* for 6 min and resuspended in infiltration buffer (10 mM MgCl₂, 200 μM acetosyringone, 10 mM MES, 0.01% [v/v] Silwet-L77, pH 5.6). Subsequently, individual nodes of rose cuttings from the cultivar ‘Old Blush’ were immersed in the *Agrobacterium* cell suspensions and subjected to a vacuum pressure of −5 kPa twice for 60 s each time. The primer sequences used in the VIGS assays are listed in Supplementary Table S1.

### Plasmid construction

For transient luciferase activity assays, 2,000-bp promoter fragments upstream of the translational start site of *RcEXPA8* were cloned into pBGWL7 (https://vectorvault.vib.be/collection) to generate *proEXPA8:LUC* reporter constructs. For EMSAs, the full-length coding sequence of *RcMYC2* without the stop codon was inserted into pET32a with the sequence encoding a His tag. For yeast one-hybrid (Y1H) assays, the full-length coding sequence of *RcMYC2* was inserted into the pGADT7 vector (prey, with the yeast GAL4 activation domain [AD]) to generate the *RcMYC2*-AD construct; a 534-bp fragment encompassing the G-box motif in the *RcEXPA8* promoter was cloned into the pHIS2 vector to generate the *proRcEXPA8*-pHIS plasmid.

To analyze protein–protein interactions by split LUC complementation assays, the full-length coding sequences of *RcMYC2* and *RcMED25* without stop codon were individually inserted into the pMK7WG-nL-2 (nLUC) or pMK7WG-cL-2 (cLUC) vector (https://vectorvault.vib.be/collection) by LR reaction. For yeast two-hybrid (Y2H) assays, the full-length coding sequences of RcMYC2 and RcMED25 without the stop codon were cloned into the pGADT7 vector (prey, AD) or the pGBKT7 vector (bait, BD) to generate the RcMED25-AD and RcMYC2-BD constructs.

### Luciferase reporter assays

For dual-luciferase reporter assays, mixtures of Agrobacterium cell suspensions each harboring the *LUC* reporter gene driven by the *RcEXPA8* promoter or an effector plasmid overexpressing *RcMYC2*, *RcMED25*, or *RcHDA8* were co-infiltrated into the leaves of *N. benthamiana* plants. For split-luciferase complementation assays, mixtures of Agrobacterium cell suspensions each carrying a construct encoding an nLUC or cLUC fusion to RcMYC2, RcMED25, or RcHDA8 were co-infiltrated into *N. benthamiana* leaves. Following incubation in a 25 °C growth chamber for 48–72 h, infiltrated areas were sprayed with 1 mM D-luciferin sodium salt (Yeasen, New York, USA) and incubated in darkness for 10 min. Luminescence was imaged using a CCD camera (Andor Technology, https://andor.oxinst.com/). The primers used in this study are listed in Supplementary Table S1.

### Yeast assays

For the Y1H assay, the CDS of *RcMYC2* was cloned into the pGADT7 vector, and the promoter fragments of *RcEXPA8* were cloned into the pHIS2 vector. According to the manual of the Yeastmaker Yeast Transformation Kit (Clontech, Mountain View, CA, USA), the two recombinant plasmids described above were co-transformed into Y187 yeast competent cells, which were then co-cultured on -Trp/-His/-Leu selective medium supplemented with 3-amino-1,2,4-triazole (3-AT). The concentration of 3-AT required to prevent the growth of Y187 yeast cells by inhibiting the self-activation of the pHIS vector on -Trp/-His selective medium was determined. Protein–protein interactions were detected on -Trp/-His/-Leu selective medium containing 3-AT at the optimized concentration. The primer sequences used in the Y1H assay are listed in Supplementary Table S1.

For the Y2H assay, the appropriate pairs of *RcMYC2*-AD or *RcHDA8*-AD and *RcMED25*-BD plasmids, or the empty vectors, were co-introduced into the *S. cerevisiae* strain Y2H Gold using the same kit as above. Positive transformants were identified by growth on SD/− Leu/− Trp medium; these were respotted onto fresh SD/− His/− Leu medium and SD/− His/− Leu/− Trp medium to identify potential interactors and onto SD/− Ade/− His/− Leu/− Trp medium to validate interactions. The primer sequences used in the Y2H assay are listed in Supplementary Table S1.

### Bimolecular Fluorescence Complementation (BiFC) assay

BiFC analysis was performed following the method described by Wang et al. (2025). The full-length coding sequences of *RcMYC2*, *RcMED25*, and *RcHDA8* were individually inserted into vectors carrying the sequence encoding the N-terminal (nYFP) or C-terminal (cYFP) half of yellow fluorescent protein (YFP) to generate the plasmids *RcMYC2-nYFP*, *RcMED25-cYFP*, and *RcHDA8-nYFP*. Plasmid pairs (*RcMYC2-nYFP* + *RcMED25-cYFP*, *RcMED25-cYFP* + *RcHDA8-nYFP*) were co-infiltrated into *N. benthamiana* leaves via Agrobacterium-mediated infiltration. Three days post-infiltration, YFP and mCherry fluorescence were visualized using a confocal microscope (Olympus FV3000, Japan) with excitation wavelengths of 488 nm and 561 nm, respectively. The primer sequences used in the BiFC assay are listed in Supplementary Table S1.

### Pull-down assay

The CDS of RcMYC2 and RcMED25 were inserted into the pET32a vector containing a His tag sequence, and the CDS of RcMED25 and RcHDA8 were recombined into the pGEX4T-1 vector, which harbors a GST-tag sequence. The primers used are listed in Supplementary Table S1. The recombinant plasmids were transformed into *Escherichia coli* DE3 to express the tagged proteins, which were then purified and verified by Western blotting. For the pull-down assay, the first step involved purifying the protein mixtures (RcMYC2 + RcMED25 and RcMED25 + RcHDA8) separately using a His-tagged protein purification kit (CoWin Biotech, Beijing, China; https://www.cwbio.com). Following elution, the second step was to detect the eluted products by Western blotting using anti-GST and anti-His antibodies (CoWin Biotech).

### Electrophoretic Mobility Shift Assay (EMSA)

A biotin-labeled G-box probe (3′ end) or its mutant probe was co-incubated with purified recombinant His-RcMYC2 at 24 °C for 20 min according to the instructions of the LightShift Chemiluminescent EMSA Kit (Thermo Fisher Scientific, Waltham, MA, USA). Reactions were separated by electrophoresis on 6% (w/v) native PAGE gels in 0.5 × Tris–borate-EDTA (TBE) buffer, with unlabeled G-box probes used as competitors. The primer sequences used in the EMSA are listed in Supplementary Table S1.

### Chromatin immunoprecipitation–quantitative PCR (ChIP-qPCR)

ChIP-qPCR analysis was performed using treated rose petals (1.5 g). The ChIP assay was conducted with an antibody against H3K9/K14ac (catalog no. 06–599, Millipore, Billerica, USA) following the method described by Bai et al. (2021). Preprocessed samples were divided into aliquots to which anti- H3K9/K14ac antibody and IgG were added separately. Samples with IgG served as negative controls, whereas those with H3K9/K14ac antibody were used as experimental groups. Enrichment was normalized to the H3K9/K14 acetylation level of P1. We also confirmed that there were no significant differences in H3 levels among different samples. The primer sequences used in ChIP-qPCR are listed in Supplementary Table S1.

## Supplementary Information

Supplementary Material 1. Supplemental Fig. S1. Spray with MeJA, DIECA, or water (control) at the bud visible stage until stage 1. Treatments included 100 µM MeJA, 1 mM DIECA, or water.

Supplementary Material 2. Supplemental Fig. S2. Flower size caused by heterologous overexpression of *RcMYC2* in *Arabidopsis Thaliana*. (a) Relative expression level of *RcMYC2* in *Arabidopsis* thaliana. (b) Comparison of flower organ size in *Arabidopsis*. (c) Comparison of flower length, petal length, width and aspect ratio. (d) Comparison of petal cells. ** indicated significant difference in Tukey HSD test (*p*<0.01).

Supplementary Material 3. Supplemental Fig. S3. Phylogenetic Tree Analysis of EXP gene family in *Arabidopsis thaliana* and *Rosa chinensis*.

Supplementary Material 4. Supplemental Fig. S4. VIGS interference with *RcMED25* gene in *Rosa chinensis* resulted in bigger flower size. (a) Phenotypic changes of floral organs in TRV- *RcMED25* roses. Petal phenotype comparison in TRV- *RcMED25* plants. Scale bar: 1 cm. (b)Relative expression analysis of *RcMED25* after VIGS-mediated knockdown. (c) Comparison of flower diameter and petal area. (**P < 0.01; Student’s *t*-test).

Supplementary Material 5. Supplemental Fig. S5. Phylogenetic tree analysis of HDA family genes in rose and their interaction with RcMED25. (a)Phylogenetic Tree Analysis of HDA Family Genes in *Arabidopsis thaliana* and *Rosa chinensis*. (b) Luciferase (LUC) assays confirmed the interaction between RcMED25 and five RcHDAC family members: RcHDA1/19, RcHDA3, RcHDA6, RcHDA8, and RcHDA14.

Supplementary Material 6. Supplemental Fig. S6. Original uncropped scans of representative western blots displayed in this manuscript.

Supplementary Material 7. Table S1. List of primers used.

## Data Availability

The data supporting this study are available from the corresponding author upon reasonable request.

## Abbreviations

JA: Jasmonic acid
MeJA: Methyl jasmonate
DIECA: Diethyldithiocarbamate
RT-qPCR: Reverse transcription-quantitative polymerase chain reaction
VIGS: Virus-induced gene silencing
TPM: Transcripts per kilobase of exon model per million mapped reads
TRV: Tobacco Rattle Virus
Y1H: Yeast one-hybrid
Y2H: Yeast two-hybrid
WT: Wild-type
35S: Cauliflower mosaic virus promoter
EMSA: Electrophoretic mobility shift assay
BiFC: Bimolecular fluorescence complementation
YFP: Yellow fluorescent protein
ChIP-qPCR: Chromatin immunoprecipitation followed by quantitative PCR
Ac: Acetylation
X-Gal: 5-Bromo-4-chloro-3-indoxyl-β-D galactopyranoside
LUC: Luciferase
